# A relative quantitative Methylation-Sensitive Amplified Polymorphism (MSAP) method for the analysis of abiotic stress

**DOI:** 10.1186/s12870-017-1028-0

**Published:** 2017-04-21

**Authors:** Piotr T. Bednarek, Renata Orłowska, Agnieszka Niedziela

**Affiliations:** Department of Plant Physiology and Biochemistry, Plant Breeding and Acclimatization Institute - National Research Institute, Radzików, 05-870 Błonie, Poland

## Abstract

**Background:**

We present a new methylation-sensitive amplified polymorphism (MSAP) approach for the evaluation of relative quantitative characteristics such as demethylation, de novo methylation, and preservation of methylation status of CCGG sequences, which are recognized by the isoschizomers *Hpa*II and *Msp*I. We applied the technique to analyze aluminum (Al)-tolerant and non-tolerant control and Al-stressed inbred triticale lines. The approach is based on detailed analysis of events affecting *Hpa*II and *Msp*I restriction sites in control and stressed samples, and takes advantage of molecular marker profiles generated by *Eco*RI/*Hpa*II and *Eco*RI/*Msp*I MSAP platforms.

**Methods:**

Five Al-tolerant and five non-tolerant triticale lines were exposed to aluminum stress using the physiologicaltest. Total genomic DNA was isolated from root tips of all tolerant and non-tolerant lines before and after Al stress following metAFLP and MSAP approaches. Based on codes reflecting events affecting cytosines within a given restriction site recognized by *HpaII* and *MspI* in control and stressed samples demethylation (DM), de novo methylation (DNM), preservation of methylated sites (MSP), and preservation of nonmethylatedsites (NMSP) were evaluated. MSAP profiles were used for Agglomerative hierarchicalclustering (AHC) based on Squared Euclidean distance and Ward’s Agglomeration method whereas MSAP characteristics for ANOVA.

**Results:**

Relative quantitative MSAP analysis revealed that both Al-tolerant and non-tolerant triticale lines subjected to Al stress underwent demethylation, with demethylation of CG predominating over CHG. The rate of de novo methylation in the CG context was ~3-fold lower than demethylation, whereas de novo methylation of CHG was observed only in Al-tolerant lines.

**Conclusions:**

Our relative quantitative MSAP approach, based on methylation events affecting cytosines within *Hpa*II–*Msp*I recognition sequences, was capable of quantifying de novo methylation, demethylation, methylation, and non-methylated status in control and stressed Al-tolerant and non-tolerant triticale inbred lines. The method could also be used to analyze methylation events affecting CG and CHG contexts, which were differentially methylated under Al stress. We cannot exclude that the methylation changes revealed among lines as well as between Al-tolerant and non-tolerant groups of lines were due to some experimental errors or that the number of lines was too small for ANOVA to prove the influence of Al stress. Nevertheless, we suspect that Al tolerance in triticale could be partly regulated by epigenetic factors acting at the level of DNA methylation. This method provides a valuable tool for studies of abiotic stresses in plants.

## Background

When plants are stressed, their DNA methylation patterns may change, as indicated by numerous morphological [1–3], biochemical [4, 5], and molecular [6, 7] studies. Alterations of epigenetic states play essential roles in protecting organisms from environmental stresses [7] and are believed to be involved in plant immunity [8]. Abiotic stresses such as drought [9], cold [10], salt [11], or heavy metals in the soil [12] can influence methylation patterns. Consistent with this, DNA methylation in *Poa annua* populations [13] is influenced by climatic factors, and similar evidence was obtained for *Deschampsia antarctica* [14]. Even short-term stresses affect DNA methylation, e.g., in barley regenerants derived via in vitro tissue culture from anthers [15]. In both barley [15] and *Gentiana pannonica* [16], global DNA methylation in regenerants increased relative to donor levels; in triticale, the DNA methylation of regenerants and their progeny decreased in comparison to those of donor plants and did not return to the initial level even after several reproductive cycles [17]. Furthermore, DNA methylation changes induced by abiotic stresses can be stably passed to progeny [18].

In plants, DNA methylation on cytosine occurs in all sequence contexts: symmetric CG and CHG (where H equals either A, C, or T) and asymmetric CHH. Both CG and CHG methylation can be copied during DNA replication, whereas CHH methylation must be reestablished in each generation via mechanisms involving DRM1, DRM2, and iRNAs [8]. DNA methylation patterns may change in response to stressful conditions [6], and such changes in symmetric CG sites may be involved in the regulation of gene expression [19, 20]. By contrast, methylation of CHG and CHH is linked to regulation of transposon activity via chromatin remodeling [21–23] in response to environmental factors.

Many approaches are available for quantitative analysis of global genome DNA methylation. Probably the simplest and the most robust of these is based on RP-HPLC [24]. Recently, the methylation-sensitive amplified fragment length polymorphism (metAFLP) method was introduced [15] and then extended [25] to enable quantification of changes in sequence and DNA methylation sites (i.e., de novo methylation and demethylation) in a single experiment. Another option is methylation-sensitive amplified polymorphism (MSAP), which is based on AFLP technology and takes advantage of two isoschizomers that recognize the same restriction site [26]. This approach was adapted to the analysis of DNA methylation pattern in plants [27] and successfully used to study changes in cytosine methylation under abiotic stresses [11, 28–30]. Using this technique, changes in DNA methylation sites could be evaluated by comparing molecular patterns [9]. Consensus has not been reached regarding how best to interpret MSAP outputs [31, 32], and at least five alternatives have been proposed [33]. However, none of the options takes into account the multiple events (reflecting various methylation pattern states or changes) that must take place simultaneously to explain the differences in individual digestion patterns between control and stressed materials. In addition, because mutations are hardly difficult to identify using the MSAP approach, they are excluded from the underlying models [33]. Further confusing interpretation of the results, no systematic studies have investigated the dependence of *Hpa*II and *Msp*I activities on the cytosine methylation state at all dCs in dsDNA [31]. Thus, a detailed analysis of the effects of methylation changes at restriction sites recognized by *Hpa*II and *Msp*I endonucleases would allow the potential of MSAP to be more fully realized. Such an analysis would need to incorporate an assessment of the background changes at restriction sites caused by abiotic stresses.

The aim of this study was to develop a theoretical model for quantification of cytosine methylation status at restriction sites of the isoschizomers *Hpa*II and *Msp*I, using only the documented activities of these enzymes, based on the MSAP profiles of triticale plants grown under Al stress.

## Results

**Fig. 1:**
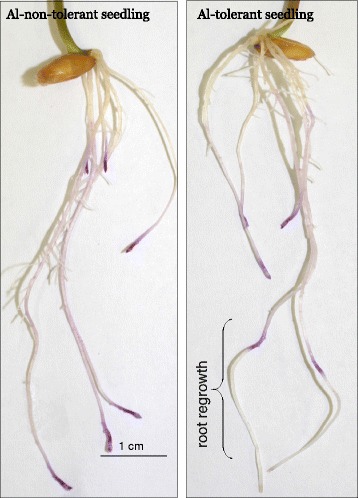
Aluminum tolerance test according to Anioł A [54]. Roots were examined 48 h after staining with Eriochrome Cyanine R

Physiological tests revealed that root regrowth of five Al-tolerant triticale lines (T1, T2, T3, T4, and T5) ranged from 0.8 to 2.8 cm in the presence of Al^3+^ ions, whereas no regrowth was observed in five non-tolerant lines (NT1, NT2, NT3, NT4, and NT5) (Fig. 1).

Analysis of documented methylation patterns of the restriction site recognized by *Hpa*II and *Msp*I showed that *Hpa*II can cut at sites that are either non-methylated or contain one methylated external cytosine, whereas *Msp*I cuts non-methylated sites and those with one or two methylated internal cytosines [26]. Comparison of the digestion patterns of these isoschizomers could be used to evaluate the extent of restriction site DNA methylation. The original MSAP approach can be used to analyze abiotic stresses when identical samples are analyzed under different conditions (i.e., control and stressed) [11, 28, 29]. In such cases, the presence or absence of molecular markers generated by *Eco*RI/*Hpa*II and *Eco*RI/*Msp*I digestion in control and stressed samples can be represented by 16 four-bit codes. Each code, reflecting a specific banding pattern, has its own rationalization, as depicted in Table 1. Four molecular patterns could be easily explained by comparison of control and stressed restriction sites: code (1111) reflects the situation in which all non-methylated cytosines of a given restriction site present in controls remain non-methylated in stressed samples; (1110) indicates de novo methylation of the external cytosine in stressed samples; (1010) reflects preservation of the external mC and lack of methylation of the other C; and finally (1011) indicates demethylation of the external C and preservation of non-methylated status of the remaining Cs in stressed samples. Similarly, code (0000) pertains to the situation in which a site that is fully methylated in controls is not altered in the stressed samples. However, such a pattern cannot be easily recognized unless several replicates of stressed samples are used, and the pattern is identified in at least one of them. The other codes have more complicated explanations, and could result from differences in methylation patterns of a given restriction site between control and stressed samples (Table 1). Nevertheless, all 16 codes can be scored (Table 2). Assuming that all of the events (de novo methylation (DNM), demethylation (DM), and preservation of non-methylated (NMSP) and methylated (MSP) sites) are equally probable, it is possible to estimate the number of events of a given type, corresponding to each four-bit code, considering only the known properties of the isoschizomers. By multiplying the number of individual events participating in the explanation of a given code by the number of MSAP profiles depicted by that code, followed by summation of events of the same kind and normalization of the data (expressed as percentages), it is possible to evaluate the relative quantitative characteristics of de novo methylation, demethylation, and preservation of either methylated and non-methylated cytosines at a restriction site. Additionally, one can also determine the total number of methylated and non-methylated cytosines in stressed samples (Tables 1 and 2). Because both external and internal cytosine methylation states are being analyzed, similar reasoning enables quantification of de novo methylation and demethylation events, reflecting the CHG and CG symmetric methylation states of the restriction sites recognized by *Hpa*II and *Msp*I.

**Table 1:** Theoretical background used for the explanation of de novo methylation (DNM), demethylation (DM), preservation of non-methylated sites (NMSP) and preservation of methylated sites (MSP) events based on known properties of *Hpa*II and *Msp*I isoschizomers

	Non-stressed (NS) lines		Stressed (S) lines		Background (restriction site)			Events and their quantification	Events affecting CG/CHG sequence methylation status change
	Digest/MSAP profile				NS (5′-3′/3′-5′)	S (5′-3′/3′-5′)	NS - > S		
4-digit code/events	*EcoR*I/*Hpa*II	*Eco*RI/*Msp*I	*Eco*RI/*Hpa*II	*Eco*RI/*Msp*I					
0000	0	0	0	0	^m^C^m^CGG GGC^m^C^m^	^m^C^m^CGG GGC^m^C^m^	MM/MM-> MM/MM	MSPex × 2; MSPin × 2	
							MM/MM- > M−/−M	MSPex × 2; DMin × 2	DM-CG × 2
					^m^CCGG GGCC^m^	^m^CCGG GGCC^m^	M−/−M- > M−/−M	MSPex × 2; NMSPin × 2	
							M−/−M- > MM/MM	MSPex × 2; DNMin × 2	DNM-CG × 2
0001	0	0	0	1	^m^C^m^CGG GGC^m^C^m^	C^m^CGG GGCC	MM/MM- > −M/−−	DMex × 2; DMin; MSPin	DM-CHG × 2; DM-CG
							MM/MM- > −M/M-	DMex × 2; MSPin × 2	DM-CHG × 2;
					^m^CCGG GGCC^m^	C^m^CGG GGC^m^C	M−/−M- > −M/−−	DMex × 2; DNMin; NMSPin	DM-CHG × 2; DNM-CG
							M−/−M- > −M/M-	DMex × 2; DNMin × 2	DM-CHG × 2; DNM-CG × 2
0010	0	0	1	0	^m^C^m^CGG GGC^m^C^m^	^m^CCGG GGCC	MM/MM - > M−/−−	DMex; DMin × 2; MSPex	DM-CHG; DM-CG × 2
					^m^CCGG GGCC^m^		M−/−M- > M−/−−	DMex; NMSPin × 2; MSPex	DM-CHG
0011	0	0	1	1	^m^C^m^CGG GGC^m^C^m^	CCGG GGCC	MM/MM- > −−/−−	DMex × 2; DMin × 2	DM-CHG × 2; DM-CG × 2
					^m^CCGG GGCC^m^		M−/−M- > −−/−−	DMex × 2; NMSPin × 2	DM-CHG × 2
0100	0	1	0	0	C^m^CGG GGCC	^m^C^m^CGG GGC^m^C^m^	-M/−− − > MM/MM	DNMex × 2; DNMin; MSPin	DNM-CHG × 2; DNM-CG
							-M/−− − > M−/−M	DNMex × 2; DMin; NMSPin	DNM-CHG × 2; DM-CG
					C^m^CGG GGC^m^C	^m^CCGG GGCC^m^	-M/M- - > MM/MM	DNMex × 2; MSPin × 2	DNM-CHG × 2
							-M/M- - > M−/−M	DNMex × 2; DMin × 2	DNM-CHG × 2; DM-CG × 2
0101	0	1	0	1	C^m^CGG GGCC	C^m^CGG GGCC	-M/−− − > −M/−−	NMSPex × 2; MSPin; NMSPin	
							-M/−− − > −M/M-	NMSPex × 2; MSPin; DNMin	DNM-CG
					C^m^CGG GGC^m^C	C^m^CGG GGC^m^C	-M/M- - > −M/−−	NMSPex × 2; MSPin; DMin	DM-CG
							-M/M- - > −M/M-	NMSPex × 2; MSPin × 2	
0110	0	1	1	0	C^m^CGG GGCC	^m^CCGG GGCC	-M/−− − > M−/−−	DNMex; DMin; NMSPex; NMSPin	DNM-CHG; DM-CG
					C^m^CGG GGC^m^C		-M/M- - > M−/−−	DNMex; DMin × 2; NMSPex	DNM-CHG; DM-CG × 2
0111	0	1	1	1	C^m^CGG GGCC	CCGG GGCC	-M/−− − > −−/−−	NMPex × 2; DMin; NMSPin	DM-CG
					C^m^CGG GGC^m^C		-M/M- - > −−/−−	NMSPex × 2; DMin × 2	DM-CG × 2
1000	1	0	0	0	^m^CCGG GGCC	^m^C^m^CGG GGC^m^C^m^	M−/−− − > MM/MM	MSPex; DNMex; DNMin × 2	DNM-CHG; DNM-CG × 2
						^m^CCGG GGCC^m^	M−/−− − > M−/−M	MSPex; DNMex; NMSP × 2	DNM-CHG
1001	1	0	0	1	^m^CCGG GGCC	C^m^CGG GGCC	M−/−− − > −M/−−	DMex; DNMin; NMSPex; NMSPin	DM-CHG; DNM-CG
						C^m^CGG GGC^m^C	M−/−− − > −M/M-	DMex; DNMin; NMSPex; DNMin	DM-CHG; DNM-CG × 2
1010	1	0	1	0	^m^CCGG GGCC	^m^CCGG GGCC	M−/−− − > M−/−−	MSPex; NMSPin × 2; NMSPex	
1011	1	0	1	1	^m^CCGG GGCC	CCGG GGCC	M−/−− − > −−/−−	DMex; NMSPin × 2; NMSPex	DM-CHG
1100	1	1	0	0	CCGG GGCC	^m^C^m^CGG GGC^m^C^m^	−−/−− − > MM/MM	DNMex × 2; DNMin × 2	DNM-CHG × 2; DNM-CG × 2
						^m^CCGG GGCC^m^	−−/−− − > M−/−M	DNMex × 2; NMSPin × 2	DNM-CHG × 2
1101	1	1	0	1	CCGG GGCC	C^m^CGG GGCC	−−/−− − > −M/−−	NMSPex × 2; DNMin; NMSPin	DNM-CG
						C^m^CGG GGC^m^C	−−/−− − > −M/M-	NMSPex × 2; DNMin × 2	DNM-CG × 2
1110	1	1	1	0	CCGG GGCC	^m^CCGG GGCC	−−/−− − > M−/−−	NMSPin × 2; NMSPex; DNMex;	DNM-CHG
1111	1	1	1	1	CCGG GGCC	CCGG GGCC	−−/−− − > −−/−−	NMSPex × 2; NMSPin × 2	

NS states for non-stressed (control) whereas S for Al stressed data. Putative MSAP banding patterns, as well as their respective 4-bit binary codes, are given in the left part of the table. Restriction sites of the NS and S, as well as genetic representation of the sites, are depicted in the middle followed by two columns illustrating events explaining the given code and those related to CHG and CG methylation types. ‘M’ and ‘m’ state for the methylated cytosine whereas ‘-’ for the non-methylated one; ‘in’ - internal, ‘ex’ - external cytosine. The numbers abbreviations in the last two columns reflect the number of events of the given type

**Table 2:** The arrangement of all possible events explaining the sixteen four-bit binary MSAP codes

Code	Demethylation (DM)	*De novo* methylation (DNM)	Methylation State Preservation (MSP)	Non-methylation State Preservation (NMSP)	Methylation (M = DNM + MSP)	Non-methylation (NM = DM + NMSP)	*De novo* methylation of CHG type (DNM-CHG)	*De novo* methylation of CG type (DNM-CG)	Demethylation of CHG type (DM-CHG)	Demethylation of CG type (DM-CG)
0000	2	2	10	2	12	4	0	2	0	2
0001	9	3	3	1	6	10	0	3	8	1
0010	4	0	2	2	2	6	0	0	2	2
0011	6	0	0	2	0	8	0	0	4	2
0100	3	9	3	1	12	4	8	1	0	3
0101	1	1	5	9	6	10	0	1	0	1
0110	3	2	0	3	2	6	3	0	0	3
0111	3	0	0	5	0	8	0	0	0	3
1000	0	4	2	2	6	2	1	2	0	0
1001	2	3	0	3	3	6	2	3	2	0
1010	0	0	1	3	0	3	0	0	0	0
1011	1	0	0	3	0	4	0	0	1	0
1100	0	6	0	2	6	2	4	2	0	0
1101	0	3	0	5	3	5	0	3	0	0
1110	0	1	0	3	1	3	1	0	0	0
1111	0	0	0	4	0	4	0	0	0	0

### Quantifying methylation events in Al-stressed triticale inbred lines

Of the 16 available MSAP profiles, we identified only seven in ten triticale lines that were maintained under control and Al-stressed conditions (Table 3). The most frequent were the cases corresponding to codes 1010, 0101, and 1111, whereas code 1100 was detected only twice. The patterns of the seven codes were more or less uniform among the samples analyzed.

**Table 3:** The arrangement of the profiles reflecting given MSAP four-bit binary code evaluated among control and Al stressed triticale inbred lines

4-bit code/events	NT1	NT2	NT3	NT4	NT5	T1	T2	T3	T4	T5
0010	7	8	7	8	7	9	7	8	8	8
0011	9	5	9	10	5	8	10	8	7	5
0101	142	142	142	142	142	142	142	142	142	142
1010	212	211	212	212	213	211	212	211	214	211
1011	1	2	1	1	2	2	1	2	2	2
1100	0	0	0	0	0	1	1	0	0	0
1111	67	67	67	67	68	67	67	66	68	67

When the codes were converted into events of the corresponding type (Table 4), and then expressed as normalized relative quantitative characteristics (Table 5), we observed slight differences among the analyzed lines. When stressed, two tolerant lines (T1 and T2) increased the percent de novo methylation (DNM%) of some cytosines (average, 4.19%) compared with that of other cytosines (average, 4.04%). The demethylation level of one tolerant line (T5) (5.89%) was lower than the average score (6.28 ± 0.25). By contrast, two non-tolerant lines (NT2 and NT5) exhibited reduced DM% relative to the three others (NT1, NT3, and NT4), whereas DNM% was comparable (average 4.04%) in all five lines.

**Table 4:** The arrangement of events recalculated based on data presented in Tables 3 and 2

Cases (events)^a^	NT1	NT2	NT3	NT4	NT5	T1	T2	T3	T4	T5
DM	225	206	225	235	202	228	231	224	218	206
DNM	142	142	142	142	142	148	148	142	142	142
MSP	936	937	936	938	937	939	936	937	940	937
NMSP	2217	2211	2217	2221	2219	2221	2221	2213	2228	2211
M (DNM + MSP)	1078	1079	1078	1080	1079	1087	1084	1079	1082	1079
NM (DM + NMSP)	2442	2417	2442	2456	2421	2449	2452	2437	2446	2417
DNM-CHG	0	0	0	0	0	4	4	0	0	0
DNM-CG	142	142	142	142	142	144	144	142	142	142
DM-CHG	51	38	51	57	36	52	55	50	46	38
DM-CG	174	168	174	178	166	176	176	174	172	168
Total (denomiator)	3520	3496	3520	3536	3500	3536	3536	3516	3528	3496

^a^M and NM reflect methylation and non-methylation of C within restriction site of the Al stressed materials. The detailed description of abbreviation is given in Table 1

**Table 5:** Quantitative characteristics evaluated based on MSAP data expressed in percentages

Quantitative data^a^	NT1	NT2	NT3	NT4	NT5	Average	T1	T2	T3	T4	T5	Average
NMSP%	62.98	63.24	62.98	62.81	63.40	63.08 ± 0.23	62.81	62.81	62.94	63.15	63.24	62.99 ± 0.19
MSP%	26.59	26.8	26.59	26.53	26.77	26.66 ± 0.12	26.56	26.47	26.65	26.64	26.80	26.62 ± 0.12
DM%	6.39	5.87	6.39	6.65	5.77	6.22 ± 0.37	6.45	6.53	6.37	6.18	5.89	6.28 ± 0.25
DNM%	4.03	4.06	4.03	4.02	4.06	4.04 ± 0.01	4.19	4.19	4.04	4.02	4.06	4.10 ± 0.08
M%	30.63	30.86	30.63	30.54	30.83	30.69 ± 0.13	30.74	30.66	30.69	30.67	30.86	30.72 ± 0.08
NM%	69.38	69.14	69.38	69.46	69.17	69.31 ± 0.14	69.26	69.34	69.31	69.33	69.14	69.28 ± 0.08
DM-CG%	4.94	4.80	4.94	5.03	4.74	4.89 ± 0.11	4.98	4.99	4.95	4.88	4.80	4.91 ± 0.07
DM-CHG%	1.45	1.09	1.45	1.61	1.03	1.32 ± 0.25	1.47	1.55	1.42	1.30	1.09	1.36 ± 0.18
DNM-CG%	4.03	4.06	4.03	4.02	4.06	4.04 ± 0.01	4.07	4.07	4.04	4.03	4.06	4.05 ± 0.02
DNM-CHG%	0.00	0.00	0.00	0.00	0.00	0.00 ± 0.00	0.11	0.11	0.00	0.00	0.00	0.05 ± 0.06

^a^M% and NM% state for methylated and non-methylated characteristics of C in stressed materials. The detailed description of abbreviation is given in Table 1

The other characteristics of the analyzed materials were comparable. Detailed analysis of DM-CG% (percentages of demethylated CG sequences within restriction sites recognized by *Hpa*II and *Msp*I), DM-CHG%, DNM-CG%, and DNM-CHG% revealed that DM-CHG% and DNM-CHG% were lower than their CG counterparts. Moreover, DNM-CG% was slightly lower than DM-CG%, whereas DNM-CHG% was always many times lower than DM-CHG%. It should be emphasized, however, that DNM-CHG% was determined exclusively in two tolerant lines (NT1 and NT2).

**Fig. 2:**
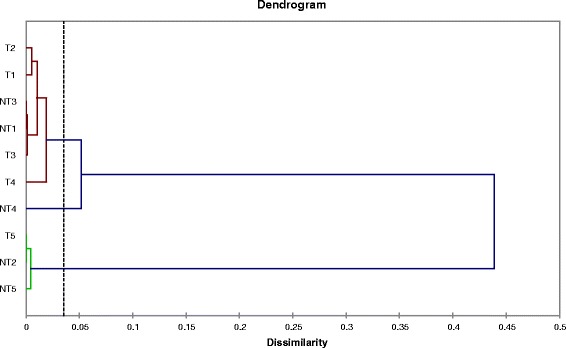
Agglomerative hierarchical clustering of T and NT lines. The *dotted line* represents the automatic truncation allowing discrimination among groups of lines

Agglomerative hierarchical clustering performed on the data presented in Table 5 demonstrated that NT1, NT3, NT4, T1, T2, T3, and T4 formed one cluster, with minor differences between T1 and T2 as well as among NT1, NT3, and T3, whereas NT2, NT5, and T5 clustered separately (Fig. 2).

Analysis of variance based on all MSAP characteristics together showed no differences among the ten lines, demonstrating that the 1% variability of the dependent variable could be explained by the explanatory variable. Similarly, ANOVA failed to detect differences between tolerant and non-tolerant lines. When individual relative quantitative MSAP characteristics were considered, they explained from 0 to 100% (in the case of DNM%) of the variance in all lines, but the differences were not significant. ANOVA performed on DNM% data between tolerant and non-tolerant lines explained 10% of the variability of the dependent variable and was negligible. When DN-CG%, DN-CHG%, DM-CG%, and DM-CHG% were used as MSAP characteristics, ANOVA also failed to identify differences among lines as well as between Al tolerant and non-tolerant groups of lines. However, up to 39% of the variability among all lines was due to differences in DN-CHG% and DM-CHG% data whereas up to 25% of the differences between T and NT lines could be explained by differences in DN-CHG%. Analysis of variance demonstrated that the differences among DN%, DM%, MP%, NMP%, M% and NM% were significant (F = 5.706, *p*-value <0.0001). Similar data were evaluated for DN-CG%, DN-CHG%, DM-CG% and DM-CHG% characteristics (F = 12.84, *p*-value <0.0001).

## Discussion

AFLP technologies (e.g., MSAP and metAFLP), which use isoschizomers with different specificities for DNA methylation at the restriction site, have been extensively exploited to analyze variations in DNA methylation patterns in plants under stress [9, 12, 15, 34]. MSAP was introduced by Reyna-Lopez et al. [26] in studies on fungi and adapted for used in plants by Xiong et al. [27], whereas metAFLP was developed for analyses of variation induced during in vitro tissue culture plant regeneration [15]. MetAFLP, which is focused on quantification of ‘dynamic’ changes that can be assessed between control and stressed materials, can accommodate both sequence and methylation changes at the same time. By contrast, MSAP was developed to study site-specific DNA methylation status. By default, in MSAP, mutations within restriction sites are hardly difficult to identify because both endonucleases are methylation sensitive [31]. Thus, such mutations are not considered in the model being assumed to be rare and not affecting the results. This assumption is supported by experiments showing that methylation at ^m^C^m^CG sequence motifs is frequent [35], whereas sequence changes are comparatively rare [36]. However, data obtained using metAFLP in triticale [18, 25] revealed that sequence changes in in vitro regenerated plants were not rare, in some cases exceeding the frequency of alterations in methylation patterns. On the other hand, similar data in barley [15] confirmed that the rate of sequence changes was significantly lower than that of changes in DNA methylation. Therefore, further studies are required to conclusively determine whether MSAP analysis is influenced by sequence changes, as well as whether such changes are species-specific. In this study, however, mutations were not incorporated into the model, following the reasoning presented by the others [29, 34–36].

In addition to the unsolved problem of point mutations, which may influence the MSAP results, it remains unclear how best to interpret the molecular profiles generated by the approach; currently, at least five alternatives exist [37]. All of them are strongly correlated, indicating that they reflect similar phenomena [32, 37]. However, the proposed interpretations do not take into account the fact that all MSAP molecular profiles reflect multiple linked events responsible for changes to, or preservation of, various methylation patterns. For example, some profiles could be explained by de novo methylation of one cytosine and demethylation of another, while the methylation status of the remaining cytosines is preserved. Moreover, the same molecular profiles could be explained by multiple types of changes in the methylation pattern. Nevertheless, by assuming that the documented activities of *Hpa*II and *Msp*I are accurate, and by analyzing the events underlying the molecular profiles generated by the MSAP approach from control and stressed samples, it is possible to quantify events that were previously invisible to established methods for MSAP data analysis. Given that our proposed method is based on an equal probability of events affecting restriction sites rather than numbers of analyzed bands, it is capable of evaluating relative quantitative characteristics such as DNM%, DM%, MSP%, and NMSP%. In that sense, our method is similar to metAFLP, where the same strategy is applied [15, 25]. It should be emphasized, however, that the method only allows strictly defined types of restriction site methylation patterns, described in the literature as recognized by *Hpa*II and *Msp*I. This assumption is likely valid because certain methylation patterns are extremely rare (5′-C^m^CGG-3′/5′-GGC^m^C-3′; 5′-CCGG-3′/5′-GGCC-3′), whereas others (i.e. 5′-^m^CCGG-3′/5′-GGC^m^C-3′; 5′-^m^CCGG-3′/5′-GGC^m^C^m^-3′; 5′-C^m^CGG-3′/5′-GGC^m^C^m^-3′), to the best of our knowledge, have never been described [31]. Thus, it would not be reasonable to incorporate them into the model. Nevertheless, we cannot exclude the possibility that, in some cases, even such rare patterns may appear. Studies of the properties of *Hpa*II and *Msp*I, including all putative methylation patterns of the restriction site, could help resolve this issue.

As stressed earlier, the proposed method assumes that there is an equal probability of events affecting restriction sites. In our earlier studies using the metAFLP approach [15, 25], we demonstrated, however, that de novo demethylation and sequence changes varied in barley [15] and triticale [25]. Thus, the equal probability assumption should be validated e.g., by a whole-genome bisulfite approach [38, 39] conducted on a given genome and restricted to restriction sites recognized by *Hpa*II and *Msp*I isoschizomers. However, despite the availability of triticale sequencing data [40], this data cannot be adopted for evaluation in a more advanced MSAP model because information about methylation changes has not been evaluated yet. If available, the information could be easily incorporated into the relative quantitative MSAP approach by using relevant probability factors that most probably will be species specific.

Moreover, the relative quantification model assumes that DNA digestion is complete and that co-migrating markers can be neglected. Thus, information obtained from a quantitative treatment of the marker band intensities) could be lost. Nevertheless, quantification of band intensities could be incorporated into the model assuming that the denser bands originate from the same event types. Quantification could be accomplished by multiplication of the events represented by more intense bands by the degree to which their intensity exceeds the average level of the given marker. However, without sequencing data, it will be difficult to be sure that putative differences in band intensities are not related to co-migrating markers of distinct origin and are not necessarily related to the same methylation change as the weaker markers. Assuming that differences in band density are relatively rare and the fact they may not necessary reflect identical events quantification of such cases was omitted by us.

The proposed MSAP approach allowed us to evaluate quantitative characteristic of ten triticale inbred lines that differed in regard to Al tolerance. Al stress resulted in elevated demethylation in both non-tolerant and tolerant plants, with de novo methylation occurring less frequently than demethylation. Total demethylation due to Al stress was about 2% in both cases. These observations are in close agreement with data obtained from salt-tolerant and non-tolerant forms of rice [37], consistent with the notion that abiotic stress results in demethylation and de novo methylation in sensitive and tolerant variants, respectively. However, this is not always the case: the opposite changes were detected in sorghum under Al stress [41], indicating that the trend could depend on the species or the specific type of stress.

The advantage of the relative quantitative MSAP approach is that it can evaluate subtle effects related to methylation events affecting CG and CHG contexts, using reasoning similar to that applied to the general characteristics. Such sequences play crucial roles in plant genomes [8, 42] because they contribute to the symmetric methylation of DNA. Symmetric and asymmetric methylation may predominate in different regions of the genome, such as genes and transposable elements (TEs), as well as in their up and downstream regions [43]. Moreover, symmetric methylation is associated with either fixed or recent changes in methylation patterns [7, 44]. Changes in DNA methylation affecting CG, CHG, and CHH contexts are involved in adaptive responses to abiotic stresses [43, 45, 46]. Such alterations could be of value when determining stress levels in analyzed materials [19, 46, 47]. Therefore, the development of a relatively inexpensive marker-based approach would be a valuable alternative to whole-genome bisulfite sequencing [48–50].

Although the results of relative quantitative MSAP, which reveals differences in methylation status of restriction sites between control and stressed samples, are generally in good agreement with metAFLP and HPLC data [17], they do not reveal what happens in the CG and CHG contexts. In the triticale lines we studied, CG sequences underwent most of the changes in methylation status in response to Al stress, with DM% slightly exceeding DNM%. Similarly, DM% was higher than DNM% in CHG, but the overall level of changes in this context was usually 3-fold lower than in CG. In addition, in both tolerant and non-tolerant lines, demethylation of CG and CHG exceeded de novo methylation. Only in two Al-tolerant lines, we observed an increase in de novo methylation of CHG sequences. In general, our data are congruent with those for tobacco [19], in which Al stress results in demethylation of CG sequences within coding regions. However, we observed no effects in the CHG or CHH context, regardless of the stress [19]. Demethylation of CG and CHG sequences was also observed in cotton under alkali stress [28]. Similar demethylation of symmetric and asymmetric sequences within promoter regions was observed in wheat [46], whereas in *Chloris virgata*, de novo CHG methylation under salt and alkaline stresses occurred in roots without an accompanying increase in CG methylation [45]. On the other hand, in sorghum, Al stress induces de novo methylation of CG or CHG sequences, depending on the line [41]. Thus, it is possible for methylation changes to affect symmetric and asymmetric contexts in different directions, and detailed studies are required to understand this phenomenon and its role in abiotic stresses.

The results of relative quantitative MSAP revealed that the analyzed triticale lines could differ from each other with respect to DNA methylation patterns induced by Al stress. Two of the non-tolerant lines and one tolerant line were distinct from the other samples (see also dendrogram Fig. 2), and in particular exhibited lower levels of demethylation. It has been suggested that changes in methylation pattern are to some extent stochastic [47, 51, 52]. On the other hand, the high degree of similarity among the Al-tolerant lines suggest that in any given case methylation could be directed towards specific genomic regions. This notion is supported by the observed increase in CHG de novo methylation in Al-tolerant lines. However, these changes were rather small and limited to a few lines. Moreover, hierarchical clustering used for the visualization of putative differences among triticale lines could be the result of chance, which could lead to misinterpretation of the data. Putative misinterpretation of the results of cluster analysis seems to have been confirmed by ANOVA, which failed to reveal differences between Al tolerant and non-tolerant triticale lines based on any relative quantitative MSAP characteristic. Alternatively, analysis of variance may suggest that the number of lines was too small to discriminate between the analyzed materials at the level of methylation changes affecting CCGG restriction sites recognized by the *Hpa*II and *Msp*I endonucleases used in the MSAP approach. Thus, it remains unclear whether the methylation differences reflect biologically relevant phenomenon not univocally supported by statistics or experimental errors. Interestingly, however, ANOVA demonstrated significant differences among all MSAP characteristics, suggesting that they may reflect biologically important phenomenon. Evidently, further studies are needed to determine whether effects on DNA methylation patterns as subtle as those observed for CHG could be associated with the Al tolerance. On the other hand, known Al-tolerance QTLs explain up to 30% of the phenotypic variance in triticale [53], whereas the remaining 70% is unexplained. Thus, our data may suggest that epigenetic factors may also participate in Al tolerance in triticale.

Finally, it is good experimental practice to validate any approach using alternative methods [32, 39]. The results of the relative quantitative MSAP approach could be validated by comparing them to those obtained by RP-HPLC [32]. This type of analysis was performed previously by us in the case of the metAFLP analysis [17, 18], and some correlation between methylation parameters was detected by the two methods. In general, the metAFLP approach underestimated global changes. The incongruence was explained by the fact that metAFLP analyzes site DNA methylation changes affecting restriction sequences recognized by *Acc65*I and *Kpn*I isoschizomers whereas the RP-HPLC approach detects global methylation changes. Moreover, RP-HPLC is robust whereas characteristics evaluated in the case of Al tolerant and non-tolerant lines were relatively small. Thus, RP-HPLC seems to be inappropriate for the validation of most MSAP characteristics (at least in the case of our experimental data) because it delivers only limited and averaged information on DNA methylation changes. Alternatively, MSAP results could be validated by whole-genome bisulfite sequencing data [38]. Such an analysis would be beneficial for the evaluation of additional factors that could be incorporated into the MSAP approach, and would allow testing of the assumption of equal probability of methylation changes assumed in the present study. Future studies should allow validation of the MSAP approach as well as its further development via the incorporation of whole-genome sequencing data. Thus, in the case of preliminary studies where e.g., abiotic factors are suspected of affecting stressed plants the relative quantitative MSAP approach could be the method of choice.

## Conclusions

Our proposed MSAP approach is capable of quantifying events such as de novo methylation, demethylation, methylation, and preservation of methylated status within *Hpa*II–*Msp*I restriction sites. Moreover, it can quantify events affecting CG and CHG sequences, providing important insight into the role of epigenetics of abiotic stresses in plants.

The quantitative results clearly demonstrated that in triticale, Al stress resulted in demethylation and de novo methylation in both tolerant and non-tolerant triticale lines; de novo methylation of CHG sequence was affected in tolerant lines but not in non-tolerant lines. MSAP analysis was partially successful in differentiating the triticale lines. However, ANOVA showed that differences among lines as well as differences between Al tolerant and non-tolerant groups of lines based on global and individual MSAP characteristics (including CHG% and CG%, DN%, and DM%) were insignificant. This result could have been due to experimental error or to the fact that methylation changes between Al tolerant and non-tolerant lines evaluated by MSAP are not necessarily related to the abiotic stress analyzed in this study. Alternatively, the number of lines used was insufficient to perform statistical analysis on the relatively weak differences revealed by this MSAP method.

## Methods

### Plant material and growing conditions

Five Al-tolerant (T1, T2, T3, T4, and T5) and five non-tolerant (NT1, NT2, NT3, NT4, and NT5) triticale lines were exposed to aluminum stress using the physiological test described previously by Anioł [54]. Seeds were surface-sterilized by soaking in 10% sodium hypochlorite for 10 min, followed by three washes with deionized water and germination on moist filter paper in Petri dishes for 24 h. Seeds representative of each triticale line were sown on two individual polyethylene nets floated in a tray filled with basic medium containing 2.0 mM CaCl_2_, 3.25 mM KNO_3_, 1.25 mM MgCl_2_, 0.5 mM (NH_4_)_2_SO_4_, and 0.2 mM NH_4_NO_3_ (pH 4.5). Seedlings were maintained in a controlled-environment growth cabinet (POL-EKO-APARATURA, ST500 B40 FOT10) at 25 °C, with a photoperiod of 12 h/12 h day/night, light intensity of 40 W m^−2^, and aeration. After 3 days, one of two nets representing each line was transferred for 24 h onto the same medium supplemented with 20 ppm AlCl_3_. Then, stressed seedlings were washed for 2–3 min in running water and transferred to nutrient solution without Al for 48 h. Finally, 7-day old seedlings were removed from control and stressful nutrient solutions and washed. Root tips (3–4 mm long) of control and stressed plants were cut off and kept at −70 °C prior to DNA isolation.

### DNA isolation

Total genomic DNA was isolated from root tips (3–4 mm long) using the Plant DNeasy MiniKit (Qiagen), followed by spectrophotometric quantification and integrity testing by 1.0% agarose gel electrophoresis.

### Methylation-sensitive amplification polymorphism (MSAP) profiling

The MSAP procedure is based on the metAFLP method [15]. However, instead of the *Acc*65I and *Kpn*I endonucleases, *Hpa*II and *Msp*I were used according to Xiong et al. [27]. Briefly, genomic DNA samples were digested with *Hpa*II/*Eco*RI or *Msp*I/*Eco*RI (New England Biolabs, Beverly, MA, USA). Digestion mixtures contained 5.0 U *Hpa*II or *Msp*I, 5.0 U *Eco*RI, 1× ligation buffer, 50 nM NaCl, and 0.5 mg/ml BSA. Digestion, as well as adapter ligation and pre-selective and selective PCR, were performed as described for metAFLP [15]. The sequences of *Eco*RI and *Hpa*II/*Msp*I adapters and pre-selective and selective primers were as described in Xiong et al. [27]. In order to reduce the number of fragments to be amplified by each selective primer, the primer contained selective nucleotides at its 3′-terminal end. Fourteen primer combinations with three selective nucleotides for the *Eco*RI ends and three or four selective nucleotides for the *Hpa*II-*Msp*I ends were used (HpaII-MspI/EcoRI: TTG/ACG, TAC/AAG, TTC/AAC, TGA/AGG, TCG/ACT, TGC/AGT, TGT/ATT, TCA/ATC, TCG/AGA, TGC/ACA, TTGC/AGC, TCAA/ACC, TCAA/ATG, TCAA/ATT). Each *Hpa*II–*Msp*I selective primer was end-labeled with 10–30 μCi γ-[^32^P] ATP using T4 polynucleotide kinase prior to performing PCR. The denatured PCR products were separated on 7% denaturing polyacrylamide gels and exposed to X-ray film overnight at −70 °C. MSAP analysis was performed twice for each primer combination.

### Methylation pattern analysis

#### MSAP

MSAP markers were evaluated for all triticale inbred lines (including controls and their Al-stressed counterparts). MSAP profiles were recorded as 1/0 binary matrices, where 1 indicates the presence and 0 the absence of a given fragment. The resultant code, expressed as four binary digits (bits), describes the presence/absence of each fragment in the *Eco*RI/*Hpa*II and *Eco*RI/*Msp*I digests of the control materials and their Al-stressed counterparts. Theoretically, 16 permutations are possible for a binary code with four positions. The number of identical codes was summarized and presented in a form of a table (Table 1). Each code reflects a set of events affecting cytosines within a given restriction site recognized by *Hpa*II and *Msp*I in control and stressed samples. Demethylation (DM), de novo methylation (DNM), preservation of methylated sites (MSP), and preservation of non-methylated sites (NMSP) are the events that must be considered simultaneously.

The two isoschizomers digest methylated sites differently [31, 55, 56] depending on which cytosines are or are not methylated. For example, code (1111) represents a fragment unaffected by Al stress: the four cytosines in questions are non-methylated in both the control and stressed samples from that line. Similarly, code (1110) could reflect the preservation of methylation at an external position, or non-methylation of an internal position and de novo methylation of the other external cytosine. In both instances, to interpret the code, it is necessary to take into account four independent events. The other codes can be interpreted according to similar logic (Table 1). The total number of events [related to de novo methylation (DN), demethylation (DM), and preservation of non-methylated (NMSP) and methylated (MSP) sites] was calculated as the sum of all codes reflecting the given event type, where each code was multiplied by the number of corresponding events (Table 2). The sum of all events related to all 16 codes was calculated and used as the denominator for quantitative calculations. Each event type was then expressed as a percentage.

Eventually, it may become possible to quantify de novo methylation and demethylation of cytosines at symmetric CHG and CG sites. For this type of analysis, the methylation of cytosines in the CHG context was assumed when the external nucleotide in the 5′-CCGG-3′ sequence underwent change. When the internal cytosines of the restriction sites were affected the CG methylation was assumed. To quantify both cases, the denominator described above was used, and the results were expressed as percentages. A full interpretation of all codes is given in Table 1.

### Statistical analysis

Agglomerative hierarchical clustering (AHC) based on Squared Euclidean distance and Ward’s Agglomeration method was performed in XlStat version 2015.6. ANOVA was performed using the Best model (adjusted R2) variable selection method, with a fixed intercept, a confidence interval equal to 95%, 0.0001 tolerance, and random validation settings in XlStat version 2015.6.

## Abbreviations

Al: Aluminum
DM: Demethylation
DNM: De novo methylation
MSAP: Methylation sensitive amplification polymorphism
MSP: Preservation of methylated site
NMSP: Preservation of non-methylated site
NT: Non-tolerant triticale line
T: Tolerant triticale line
